# Wilson’s Disease: A Prevalence Study in a Portuguese Population

**DOI:** 10.7759/cureus.43718

**Published:** 2023-08-18

**Authors:** Bebiana Sousa, Pedro Magalhães, Alfredo Pinto, Eunice Trindade, José Presa Ramos, Sara Freitas, Susana Lopes, Henedina Antunes

**Affiliations:** 1 School of Medicine, University of Minho, Braga, PRT; 2 Paediatrics Department, Centro Materno-Infantil do Norte do Centro Hospitalar Universitário de Santo António, Porto, PRT; 3 Internal Medicine Department, Centro Hospitalar de Vila Nova de Gaia / Espinho, Vila Nova de Gaia, PRT; 4 Internal Medicine Department, Unidade Local de Saude do Alto Minho, Viana do Castelo, PRT; 5 Paediatric Gastroenterology Department, Centro Hospitalar Universitário de São João, Porto, PRT; 6 Hepatology Unit of Internal Medicine Department, Centro Hospitalar de Tras-os-Montes e Alto Douro, Vila Real, PRT; 7 Internal Medicine Department, Hospital Senhora da Oliveira Guimarães, Guimarães, PRT; 8 Gastroenterology Department, Centro Hospitalar Universitário de São João, Porto, PRT; 9 Life and Health Sciences Research Institute - ICVS/3B's Associated Laboratory and School of Medicine, University of Minho, Braga, PRT; 10 Paediatric Gastroenterology, Hepatology and Nutrition Unit, Hospital de Braga, Braga, PRT

**Keywords:** pediatrics, internal medicine, epidemiology, genetic disorder, copper metabolism

## Abstract

Introduction

Wilson's disease (WD) is a rare and underdiagnosed genetic disorder caused by anomalous tissue copper deposition, and for which epidemiological studies, specifically in Portugal, are scarce.

Objectives

This study aimed to evaluate the prevalence and incidence of WD and provide a description of its main clinical and laboratory features.

Methods

A retrospective study was carried out, with a search between 1995 and 2015, of all patients with a minimum follow-up of three months and birth confirmed in the northern region of Portugal, with an estimated population of 3,689,682 inhabitants. Database collection was based on the Portuguese National Health Service's clinical coding system, relying on clinical data from 13 northern Portuguese hospitals, liver biopsy histology results, and hospital prescription records. Clinical and biochemical correlations were statistically assessed using chi-square, Mann-Whitney U, Friedman, and Wilcoxon tests.

Results

Over the 20-year period, a prevalence of 1:37.000 and an incidence of one per million person-year was found. A total of 94 patients were analyzed, with a slight male predominance (53%), the majority with the onset of clinical manifestations in pediatric age (56%), with a median age at diagnosis of 16.6 years (interquartile range of 12.3-20,.8 years). Most patients presented with predominant liver disease (54.8%), with more than a third with cirrhosis; mixed hepatic and neurological manifestations in 17.9%; and mainly neurological symptoms in 10.7% of the patients. Neurological impairment was strongly associated with delayed development of the manifestations of the disease (p = 0.001) and also a higher detection of Kayser-Fleischer rings (p < 0.001), present in 27.0% of the patients. Regarding therapy, penicillamine has been the most widely used, with adverse reactions reported in 24.8%. At six and 12 months after initiation of therapy, a significant decrease in liver enzymes was found (ALT: p = 0.002; AST: p = 0.002, respectively), but no significant reduction was observed in urinary copper excretion.

Conclusion

This was one of the first studies regarding WD prevalence in a Portuguese population, contributing to a better understanding of the epidemiology, diagnosis, and management of WD in the northern region of Portugal. WD should be considered in any individual with unexplained hepatic or neurological manifestations, and initial symptoms may manifest at an early age, even in children less than five years old. A high percentage of patients were identified in the early stages of the disease by asymptomatic elevation of transaminases. Following copper chelation therapy, cytolysis markers appear to be more sensitive indicators of treatment response.

## Introduction

Wilson’s disease (WD) is an inherited disorder caused by excessive copper tissue and organ deposition [1]. The ATP7B gene is responsible for a P-type adenosine triphosphatase, whose expression in hepatocytes leads to copper transportation into the trans-Golgi network where it is either incorporated into apoceruloplasmin for the synthesis of functional ceruloplasmin or secreted into bile [2-4]. Unless appropriate treatment is started, WD is characterized by a rapid and progressive natural disease history and even death. A failure in diagnosis is the most common cause of death in these patients [5]. Global prevalence is estimated to be 1:40.000 with an incidence of 1:30.000, although recent studies suggest possible underdiagnosis [6,7]. There are few studies regarding epidemiologic information on WD [8-10]. In Portugal, particularly, the largest published cohort to date is 24 patients in one tertiary care hospital [11].

The first objective of the study was to evaluate the prevalence and incidence of WD in the northern region of Portugal based on clinical records from multiple hospital centers. Secondary goals focused on the identification of the main clinical presentation forms and comparison of the parameters of copper metabolism, presence of other signs and symptoms, treatments, Ferenci score at the time of diagnosis, liver enzymes, urinary copper excretion, and liver transplantation.

This article was previously presented as an abstract at the European Society for Paediatric Gastroenterology, Hepatology and Nutrition (ESPGHAN) 50th Annual Meeting in May 2017. No conflicts of interest were declared.

## Materials and methods

A retrospective study was carried out, including all adult or pediatric patients, with no age restrictions, between 1995 and 2015, with a minimum follow-up of three months and birth confirmed in the northern region of Portugal. Clinical and biochemical correlations were statistically assessed using chi-square, Mann-Whitney U, Friedman, and Wilcoxon tests.

This study was approved by the Ethics Subcommittee for Health and Life Sciences of the University of Minho (approval no. SECVS 132/2015). Further approval was granted by every single ethical committee in all individual hospital centers included in this study. Patient data collection was conducted in strict accordance with institutional guidelines, Portuguese Law, and the rules of ethical conduct and good practice designated by the Convention on Human Rights and Biomedicine, the Declaration of Helsinki, the Guide to Good Clinical Practice, and the guidelines referred to in the International Ethical Guidelines for Biomedical Research Involving Human Subjects. Confidentiality was ensured through anonymization of collected information on patient records. Due to ethical considerations and Portuguese Law, genetic testing results were not collected.

The identification of cases of WD was performed using the Portuguese National Health Service's clinical coding system (GDH 275.1 - Copper metabolism disease), relying on clinical data from 13 Northern Portuguese hospitals, liver biopsy histological evaluations, and hospital prescription records. 

In this study were included all patients encoded as GDH 275.1 with a diagnostic Ferenci score ≥ 4 or individuals with WD who have only partially met the score due to insufficient records, but who have at least one of the following: pharmacological therapy with copper chelators or high copper (> 250 ug/g) in liver biopsy and typical symptoms. Exclusion criteria included individuals with inconclusive diagnostic studies. Deceased patient characterization was not possible due to insufficient clinical records.

The evaluated variables included age; gender; place of birth; age of onset of symptoms; age of diagnosis; duration of disease; family history; presence of Kayser-Fleischer (KF) rings; copper titration and histological findings in liver biopsy; time between biopsy and diagnosis; treatments and adverse effects; symptoms at diagnosis (hepatic, neurological, psychiatric, hematologic, endocrine, ocular, and renal); serum ceruloplasmin; plasma copper; urinary copper excretion; liver enzymes; liver transplantation; and duration of disease until transplantation.

The Portuguese Northern region comprises an area of 21,278 km^2^ with a current estimated population of 3,689,682 inhabitants, being one of the country’s largest population centers. The process of case identification by a clinical coding system on this population was automated with only positive findings reported. In order to determine the prevalence of the last 20 years in the northern region, a population estimate was obtained between 1995 and 2015, based on data from the Portuguese Institute of Statistics, concluding on an estimated mean population of 3,274,993 during those two decades [12-13]. As we are facing a genetic disease with potential new mutations, the entire population of the northern region was considered at risk. As such, for the incidence rate, we consider all new cases in the last 20 years and divided them by the estimated mean population during the study period. From this value, an annual incidence rate was estimated between 1995 and 2015. Statistical analysis was performed using SPSS version 22.0 (IBM SPSS Statistics for Windows, Armonk, NY). Analytical correlations were established using chi-square, Mann-Whitney U, Kruskal -Wallis, Friedman, and Wilcoxon tests, with a 95% confidence interval (p < 0.05).

## Results

Demographics

A total of 171 potential cases were identified by the electronic search with only 94 of those being confirmed cases by the inclusion criteria. Six individuals were deceased (6.4%). Initial manifestations of the disease occurred at pediatric age (< 18 years) in 55.8% (n = 48) of them, with a median age at diagnosis of 16.6 years (IQR = 12.3-20.8 years). Male patients were more prevalent, accounting for 53.2% (n = 50) of the cases. The average follow-up period was 15.2 ± 8.8 years. The youngest age at diagnosis was three years old, and the oldest was 60 years old. The main characteristics of the study population are summarized in Table 1.

**Table 1:** Descriptive analysis of the main characteristics of the sample. IQR: Inter-quartile range; SD: Standard deviation

Population characteristics	
Age at time of study (years) (median, IQR)	31 (25-40.75)
Age at diagnosis (years) (median, IQR)	16.58 (12.27-20.80)
Age of initial presentation (years) (median, IQR)	15.00 (9.50-19.00)
Male gender (%)	50 (53.2%)
Duration of disease (years) (average ± SD)	15.17 ± 8.83
Follow-up time (years) (average ± SD)	15.16 ± 8.83
Observed mortality (%)	6 (6.4%)

Incidence and prevalence 

The prevalence of WD in the northern region of Portugal over the 20-year period was one in 37,000 (0.003%). At the time of the conclusion of data collection in 2015, the prevalence stands at one per 45,000 inhabitants (0.002%), with an incidence of one per one million people per year. In the period of study, we verified an average of five diagnoses per year.

Clinical presentation at diagnosis

Most patients presented with predominant liver disease (54.8%), with 37.0% (n = 23) already at an advanced stage of liver disease compatible with cirrhosis and 26.6% (n = 25) with asymptomatic elevation of liver enzymes alanine transaminase (ALT) and aspartate transaminase (AST); mixed hepatic and neurological manifestations were observed in 17.9% (n = 15) and mainly neurological symptoms in 10.7% (n = 9).

Excluding asymptomatic patients, the median interval between symptom onset and diagnosis was 0.3 years (IQR = 0.2-1.6) for the mainly hepatic group and 0.7 years (IQR = 0.1-1.9) for the neurological group, with no statistically significant difference detected (p = 0.065).

**Figure 1:**
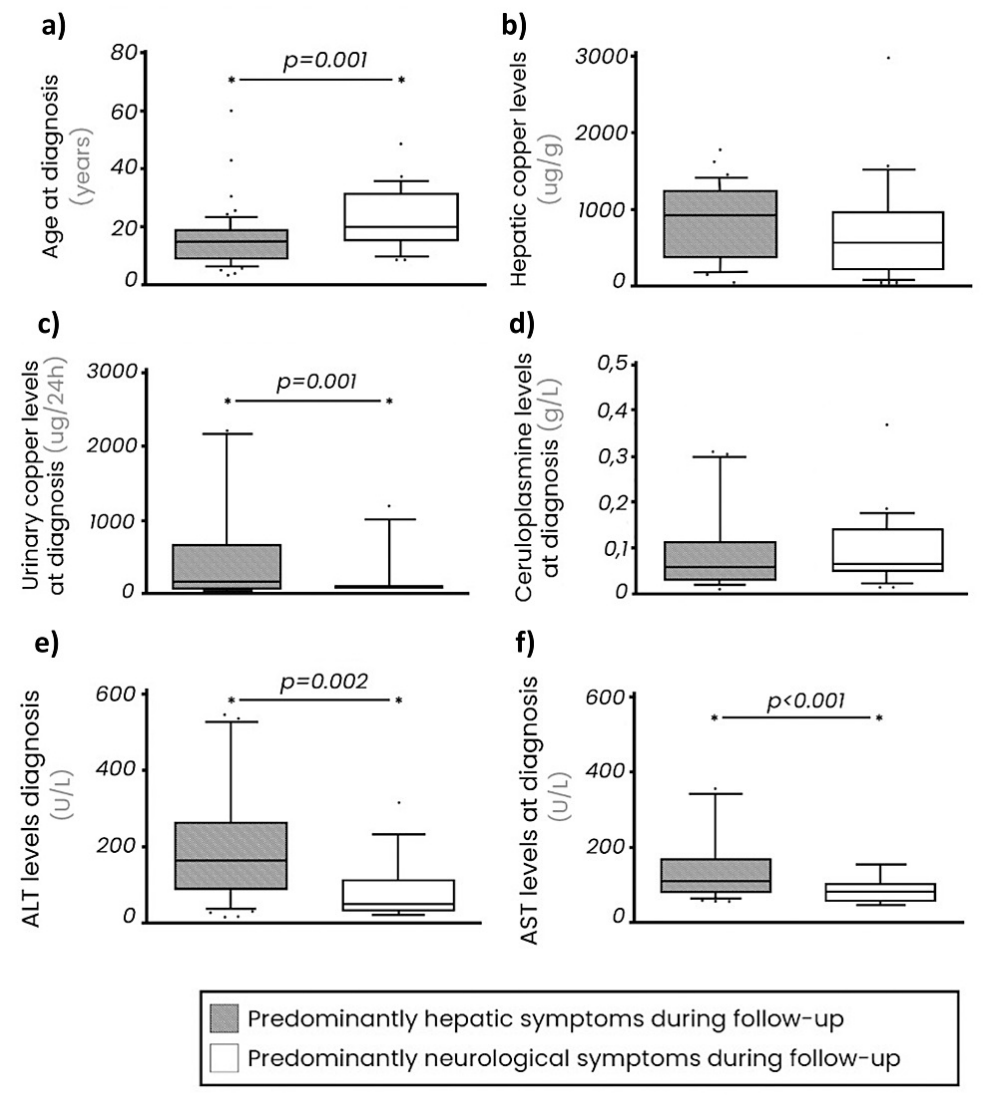
Comparison between patients presenting with predominantly hepatic and neurological symptoms at the time of diagnosis. * Significant statistical differences (p < 0.05). AST: Aspartate transaminase. ALT: Alanine transaminase.

Regarding central nervous system involvement, the main manifestations were tremors in 21.3% (n = 20), dystonia and/or speech disorders in 6.4% (n = 6), gait disturbances in 4.3% (n = 4), and psychiatric disorders in 4.3% (n = 4). Taking into account the two most frequent clinical presentations, a comparative analysis between these predominant groups was done regarding demographic, clinical, laboratory, and therapeutic characteristics. Patients with neurological presentation are significantly older at the diagnosis compared to patients with hepatic symptomatology (Z (U) = -3.365 (449); p = 0.001; r = 0.365), while with lesser urinary copper excretion levels (Z (U) = -2.32) (155); p = 0.020; r = 0.338) and liver transaminases ALT (Z (U) = -3.111 (148.5); p = 0.002; r = 0.416) and AST (Z (U) = -3.579 (126, 5), p <0.001, r = 0.474) (Figure 1). Kayser-Fleischer rings were found in 27.0% (n = 24) of WD patients and more frequently observed in adults with neurological manifestations in 79.2% (n = 19) (p < 0.001).

Analytical profile at diagnosis

Regarding analytical characteristics at diagnosis, a low serum ceruloplasmin level (mdn = 0.06 g/L; IQR = 0.03-0.14; n = 60) was found with a median urinary excretion of 84 ug/24h copper (IQR = 41-377.53; n = 47), AST 77 U/L (IQR = 49-136; n = 57), and ALT 108 U/L (IQR = 45.3-207.8; n = 56). The main analytical findings at diagnosis are summarized in Table 2. Of the 60 patients with ceruloplasmin analysis at diagnosis, seven patients had normal values.

**Table 2:** Descriptive analysis of the main analytical characteristics of the sample at diagnosis. AST: Aspartate transaminase. ALT: Alanine transaminase. IQR: Interquartile range

Main analytical findings at diagnosis	
Ceruloplasmin at diagnosis (g/L) (median and IQR)	0.06 (0.03-0.14)
Copper urinary excretion at diagnosis (ug/24 h) (median and IQR)	84 (41.0-377.5)
AST at diagnosis (UI/L) (median and IQR)	77 (49.00-136.00)
ALT at diagnosis (UI/L) (median and IQR)	108 (45.25-207.75)

Liver biopsy

Liver biopsy was performed in 57 patients (60.6%), with fibrosis and cirrhosis being the most frequently detected histopathological changes. Hepatic copper titration was performed in 56% (n = 53), with a median of 825 µg/g (IQR = 351-1205 µg/g).

Pharmacological therapy and liver transplant

**Figure 2:**
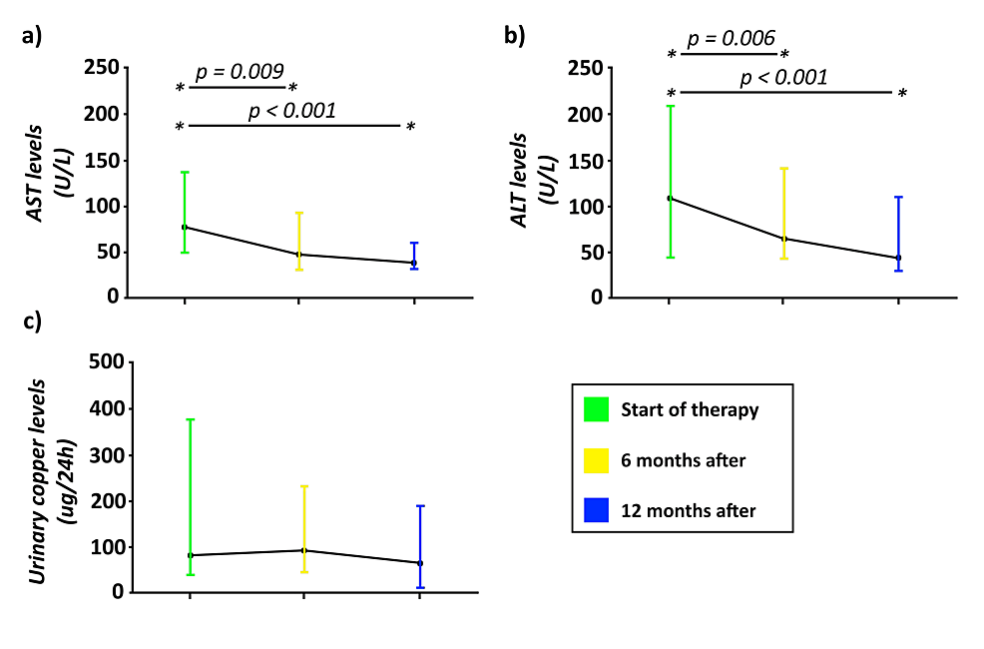
Comparison through Friedman and Wilcoxon Ranks tests of urine copper excretion, AST and ALT at diagnosis, 6 and 12 months after starting therapy. * Significant statistical differences (p<0.05). AST: Aspartate transaminase. ALT: Alanine transaminase.

Regarding therapy, penicillamine was the most frequently used, in 76.1% (n = 67) of patients, with adverse reactions observed in 28.4% (n = 25). Trientine was used in 40.9% (n = 36) of the patients at some time of the disease. Zinc therapy was used in 37.5% (n = 33). One in five of the predominantly neurological patients experienced clinical worsening after penicillamine therapy. At 6 and 12 months after initiation of therapy, a significant decrease in liver enzymes was found (ALT: p = 0.002; AST: p = 0.002, respectively), but no significant reduction was observed in urinary copper excretion (p = 0.255) (Figure 2). Liver transplant was accomplished in 23.9% (n = 21), with a median time between diagnosis and transplantation of 11 months.

## Discussion

Epidemiological data obtained, which are in line with the expected prevalence in other populations, such as in Austria, France, Taiwan, and Hong Kong, were crude disease prevalence, which has been estimated to range from 1:29,000 to 1:40,000 [8-10]. There are some specific populations with higher reported prevalence, such as Croatia (1:28,000), Sardinia (1:16,700), Israel (1:16,000), and Costa Rica (1:19,000). However, the northern region of Portugal is more in line with the estimated prevalence of 1:30,000 to 1:50,000, which appears valid for most of the USA, Europe, and Asia [10]. These determinations may, however, be underestimated in that they constitute an estimate based on a retrospective study and do not include cases wherein patients carry the disease but were never presented for testing. Although the majority of the affected population is now adults, most diagnoses were made while at pediatric age. The importance of early recognition of clinical manifestations is related to the timely initiation of therapy, leading to a better prognosis since late diagnosis is one of the most important prognostic factors leading to death [14-15].

In this study, WD presented mostly as hepatic symptoms (54.8%, n = 46) and less frequently neuropsychiatric (10.7%, n = 9), also consistent with previous studies where hepatic manifestations are reported in 45% [7,16]. As already described in the latest European Association for the Study of the Liver (EASL) guidelines on WD, hepatic manifestations are highly variable, ranging from asymptomatic to overt cirrhosis and fulminant hepatitis [16]. A liver transplant was accomplished in 23.9% (n = 21), mostly in the context of fulminant hepatitis. The age of presentation for predominantly neurological patients is described to be between 20 and 30 years of age and mainly hepatic manifestations often present between 10 and 13 years of age [16-18]. Neurological patients are, therefore, diagnosed at an older age, about 10 years later in comparison with that described in other studies and an average of five years in our sample [16].

About one-quarter of the patients presented asymptomatic elevation of transaminases, a value higher than that described in the literature, and a potential early screening method. WD should be suspected when we are faced with persistent analytical alterations without a defined etiology [19].

The predominant neurological symptom was tremors, but previous studies reported dysarthria as the main manifestation [20]. This group presented lower levels of transaminases, associated with liver damage and lower urinary copper excretion compared to with exclusive or concomitant liver manifestations [7,16]. This group of patients requires more attention regarding therapy, since, after the introduction of the classically recommended drug and most commonly used in our study, penicillamine, there is a worsening of neurological symptoms in one-fifth of the patients, superior to what has been previously described [18,21]. Therefore, there is a growing need to evaluate new effective therapies and further investigate available ones by individualizing treatment according to patient characteristics.

KF rings showed a higher prevalence in adult patients with neurological manifestations (79.2%, n = 19), which reveals a low sensitivity as an isolated diagnostic tool as other works have suggested [8,22].

WD therapy should be ad eternum, and monitoring for compliance, adverse reactions, and efficacy is essential. In this study, most patients had a statistically significant normalization of analytical parameters, such as AST and ALT, from six to 12 months after treatment. It will be of future interest to study the individual effectiveness of the different therapeutic options used in this population.

The 24-hour urinary copper excretion is a recommended analysis in the EASL guidelines for assessing the adequacy of treatment and compliance [16]. In our study, however, we found that, in the first 12 months, after the introduction of copper chelation therapy, cytolysis markers were more sensitive indicators of treatment response. Cytolysis markers appear to be good indicators of treatment response, and studies should be undertaken to compare with urinary copper excretion measurement.

Study limitations related to data collection stem from the mandatory need for hospitalization, mostly in the context of liver biopsy or clinical decompensation to obtain coding, and are dependent on adequate reporting. Enrolled patients were limited to those born in the northern region of Portugal and do not include people who moved outside the region and were diagnosed elsewhere. We also identified coded individuals without clinical evidence of pathology. Information collected from the database was dependent on the quality and availability of records, as well as provider knowledge of the disease. The variability in the quality of the information collected constituted a limitation, which was minimized by using different sources of information. Prevalence and incidence were based on medical records and were not controlled or randomized, and diagnostics were not based on systematic screening in the context of prospective studies. Deceased patient clinical information was not collected, even after testing positive as access to clinical records was severely limited and, therefore, not included.

## Conclusions

These findings contributed to a better understanding of the epidemiology, diagnosis, and management of WD, which showed to be similar to previous reports of other countries. The first clinical manifestations in the majority of WD patients occurred at pediatric age. Therefore, measures should be taken in order to establish an early diagnosis, even in children less than five years old, as evidenced by disease diagnosis as early as three years of age in our study. A high percentage of patients were identified in the early stages of the disease by asymptomatic elevation of transaminases. Although the spectrum of clinical manifestations was variable, the majority had hepatic and, to a lesser extent, neurological involvement, the latter being of later onset and diagnosis, and better methods are needed to make an earlier diagnosis. WD should be considered in any individual with unexplained hepatic or neurological manifestations. Moreover, this study showed that, in the early stages after the introduction of copper chelation therapy, cytolysis markers were more sensitive indicators of treatment response.
