# Correction: Wilson’s Disease: A Prevalence Study in a Portuguese Population

**DOI:** 10.7759/cureus.c142

**Published:** 2023-11-02

**Authors:** Bebiana Sousa, Pedro Magalhães, Alfredo Pinto, Eunice Trindade, José Presa Ramos, Sara Freitas, Susana Lopes, Henedina Antunes

**Affiliations:** 1 School of Medicine, University of Minho, Braga, PRT; 2 Paediatrics Department, Centro Materno-Infantil do Norte do Centro Hospitalar Universitário de Santo António, Porto, PRT; 3 Internal Medicine Department, Centro Hospitalar de Vila Nova de Gaia / Espinho, Vila Nova de Gaia, PRT; 4 Internal Medicine Department, Unidade Local de Saude do Alto Minho, Viana do Castelo, PRT; 5 Paediatric Gastroenterology Department, Centro Hospitalar Universitário de São João, Porto, PRT; 6 Hepatology Unit of Internal Medicine Department, Centro Hospitalar de Tras-os-Montes e Alto Douro, Vila Real, PRT; 7 Internal Medicine Department, Hospital Senhora da Oliveira Guimarães, Guimarães, PRT; 8 Gastroenterology Department, Centro Hospitalar Universitário de São João, Porto, PRT; 9 Life and Health Sciences Research Institute - ICVS/3B's Associated Laboratory and School of Medicine, University of Minho, Braga, PRT; 10 Paediatric Gastroenterology, Hepatology and Nutrition Unit, Hospital de Braga, Braga, PRT

This author list of this article has been corrected at the request of the authors to remove Ermelinda Silva, previously listed as fifth author, from the article. Dr. Silva's removal was agreed upon by the authors and was based on confusion among the author group regarding her contributions.

